# Giant cervical internal carotid artery aneurysm associated with multiple intra- and extracranial aneurysms: a case report

**DOI:** 10.3389/fsurg.2026.1936791

**Published:** 2026-08-13

**Authors:** Xavier Wong-Achi, Ambar Riley-Moguel, José Guillermo Flores-Vázquez, Alejandro Becerril-Mejía, Dora Yvette Lugo-Hilario, Edgar Nathal

**Affiliations:** Department of Neurosurgery, National Institute of Neurology and Neurosurgery, Mexico City, Mexico

**Keywords:** carotid artery aneurysm, case report, cerebral revascularization, giant aneurysm, stroke

## Abstract

The coexistence of intracranial and extracranial aneurysms is an uncommon association and suggests an underlying systemic aneurysmal disorder that involves complex therapeutic challenges. We report a young patient with multiple intra- and extracranial aneurysms, including a giant, partially thrombosed cervical internal carotid artery aneurysm causing severe hemodynamic compromise and ischemic stroke. The cervical lesion was managed with surgical trapping and aneurysmectomy without cerebral revascularization, resulting in favorable short-term clinical and angiographic outcomes. This case highlights the importance of comprehensive vascular assessment, detailed angiographic and hemodynamic assessment, and individualized decision-making regarding extracranial–intracranial bypass. Careful patient selection and detailed hemodynamic assessment may support aneurysm exclusion without bypass in selected patients with adequate collateral circulation, although long-term surveillance remains essential.

## Introduction

Extracranial internal carotid artery (ICA) aneurysms are rare vascular lesions, accounting for less than 1% of all arterial aneurysms and approximately 4% of peripheral aneurysms (1). Their clinical significance lies not only in their infrequency but also in their diverse etiologies and their potential for severe neurological complications (2). Giant or partially thrombosed cervical ICA aneurysms are particularly uncommon and pose substantial diagnostic and therapeutic challenges, as they may present with compressive symptoms, pulsatile neck masses, cranial neuropathies, or cerebrovascular ischemia due to embolization or hemodynamic compromise.

Management strategies for these lesions remain heterogeneous, ranging from open reconstruction and aneurysmectomy to endovascular treatment, depending on anatomical feasibility and the hemodynamic profile of the parent artery. Complex aneurysms often require individualized hybrid approaches or staged procedures to secure both aneurysm exclusion and adequate cerebral perfusion (2–5). The decision-making process becomes even more intricate in the presence of concomitant aneurysmal disease affecting intracranial vessels or the aorta.

We present the case of a young adult with a giant, partially thrombosed cervical ICA aneurysm causing severe flow steal and symptomatic cerebral ischemia, associated with multifocal aneurysmal disease of the bilateral ICAs and a concurrent thoracic aortic dissection. Beyond their rarity, these aneurysms may produce severe cerebral hypoperfusion, turbulent intra-aneurysmal flow, thromboembolic phenomena, and progressive collateral circulation recruitment, generating complex hemodynamic scenarios. The educational value of this case lies not only in the rarity of the vascular pathology itself, but also in the complex anatomical and hemodynamic assessment that guided surgical decision-making. This case illustrates how detailed angiographic evaluation, intraoperative anatomical findings, and collateral circulation assessment may influence the need for cerebral revascularization in selected patients with giant cervical ICA aneurysms.

## Case description

**Figure 1:**
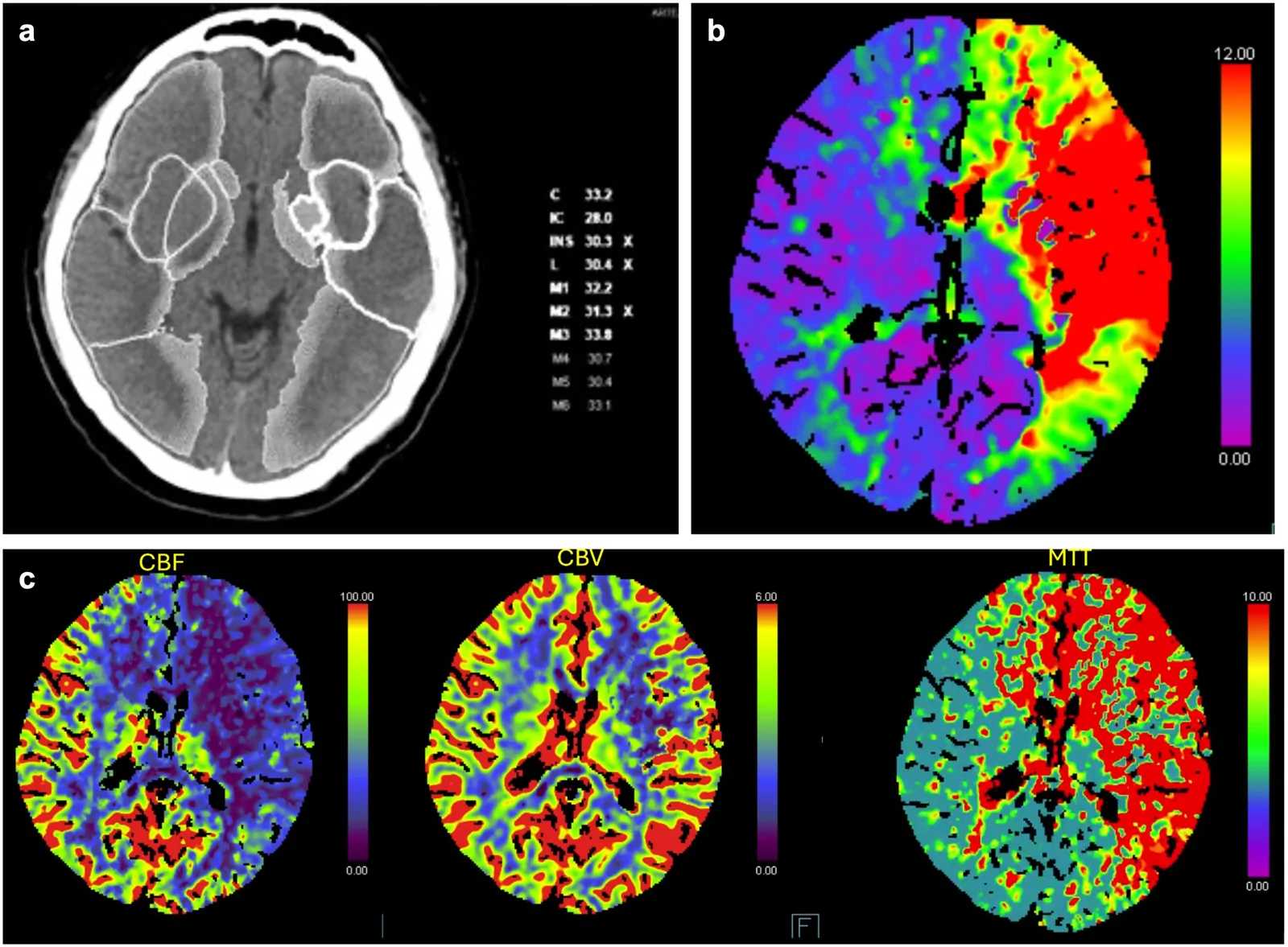
Initial non-enhanced CT and perfusion CT. **(a)** Early ischemic changes in the left MCA territory (ASPECTS 6: M2, M3, insula, lenticular). **(b)** Time-to-maximum. **(c)** Cerebral blood flow, cerebral blood volume and mean transit time, respectively. Penumbra: 385 mL. Core: 161 mL. Penumbra-Core mismatch: 2.39. The ischemic mechanism may have involved both reduced antegrade flow and thromboembolic phenomena related to the partially thrombosed aneurysm.

**Figure 2:**
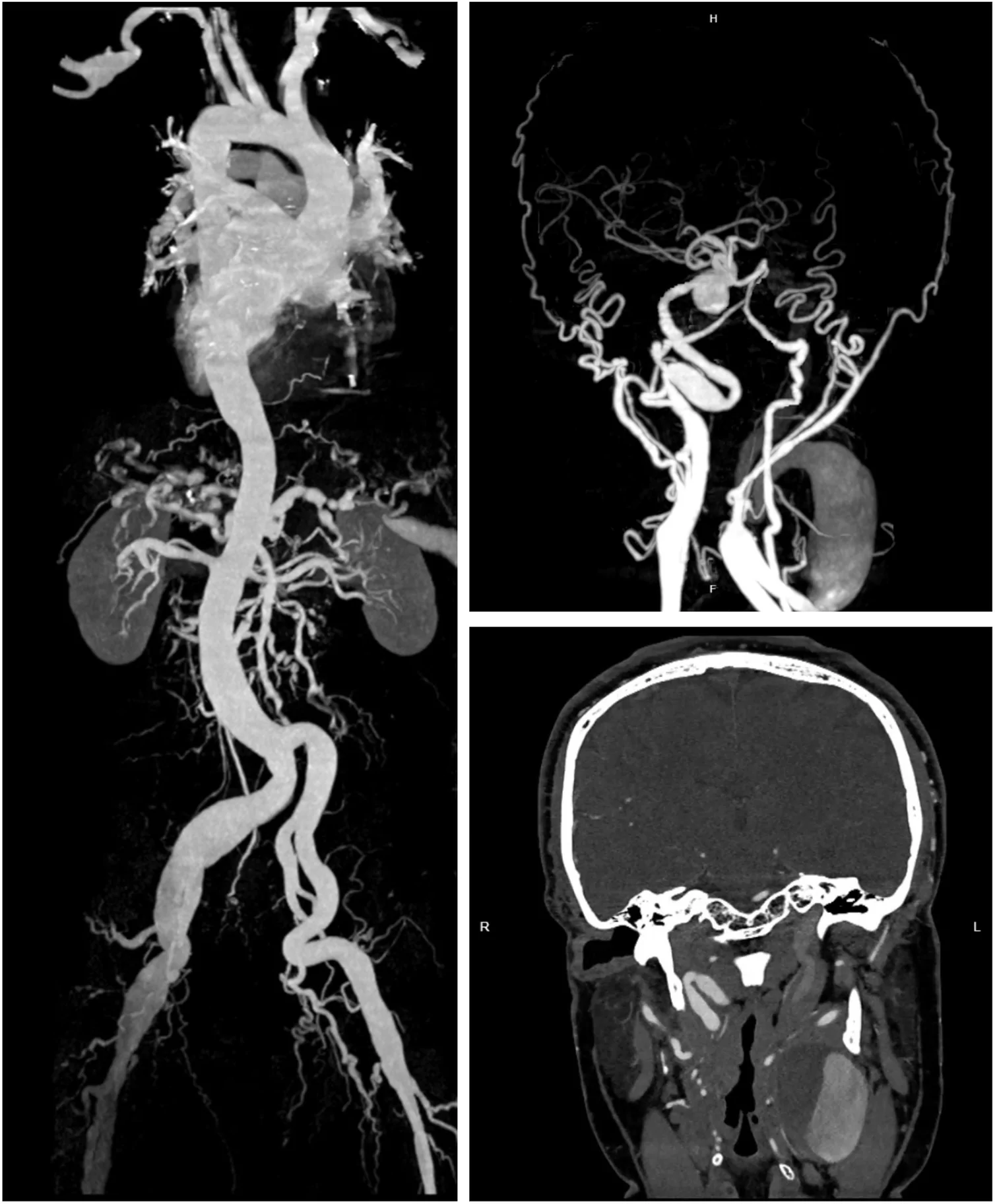
**Right:** 3D brain CT angiography reconstruction. Giant partially thrombosed aneurysm arising from the cervical segment of the left ICA, located near the carotid bifurcation, associated with marked flow reduction to the intracranial ICA. Additional findings included a large cavernous-segment aneurysm of the right ICA. **Left:** 3D Abdominal CT angiography reconstruction. Incidental thoracic aortic dissection with features of a DeBakey IIIa/Stanford B. Multiple fusiform aneurysms in the arteries of the celiac trunk and superior mesenteric artery, both common iliac arteries, and left femoral artery, plus aortoiliac dolichoectasia.

A 29-year-old man experienced a sudden loss of consciousness, remaining unresponsive for approximately one hour. On arrival at the emergency department, the patient presented with aphasia and right hemiplegia, additional physical examination revealed a firm, non-mobile left cervical mass; his mother reported that it had been present for four years and had been progressively enlarging. The patient had no known history of hypertension, smoking, recent cervical trauma, infectious disease, inflammatory or connective tissue disorder, or prior vascular intervention. No clearly documented family history of aneurysmal disease, arterial dissection, or sudden vascular death was identified during the available clinical evaluation. Due to the acute nature of the presentation, he was approached as an ischemic stroke (NIHSS 13). Non-contrast CT demonstrated early ischemic changes within the left middle cerebral artery (MCA) territory (ASPECTS 6) (Figure 1). CT angiography revealed a partially thrombosed giant aneurysm arising from the cervical segment of the left ICA, located near the carotid bifurcation, associated with marked reduction of intracranial ICA flow. Additional findings included: a dolichoectatic right ICA with a fusiform aneurysm in the cervical segment and another large aneurysm in the cavernous segment, as well as thoracic aortic dissection DeBakey IIIa/Stanford type B and other systemic multiple fusiform aneurysms (Figure 2). During the initial evaluation, the patient was not considered a candidate for intravenous thrombolysis due to the presence of a large, established ischemic core with a high risk of hemorrhagic transformation, as well as the concurrent finding of a thoracic aortic aneurysm with dissection characteristics, which represent significant contraindications for systemic thrombolytic therapy. CTA did not demonstrate a definite intracranial large-vessel occlusion amenable to mechanical thrombectomy.

**Figure 3:**
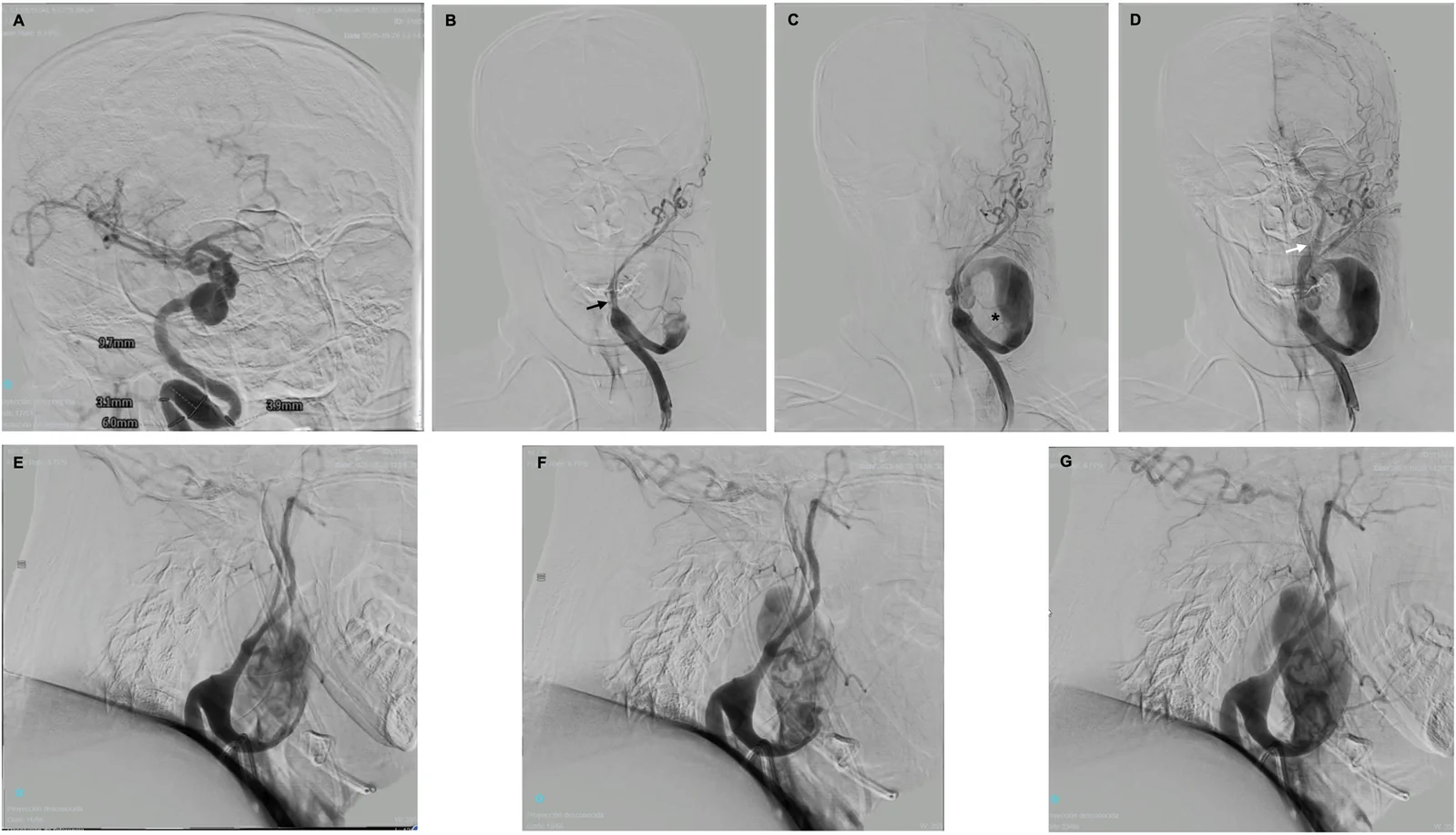
Preoperative DSA. **(A)** Oblique projection of the right carotid system demonstrating diffuse dolichoectasia with a fusiform aneurysm at C1 and a saccular aneurysm at C4–C5 (dome 12 × 14 mm; neck 5 mm) with an anterolateral projection. **(B–D)** Anteroposterior projections of the left carotid injection demonstrating a giant partially thrombosed cervical aneurysm involving the proximal ICA near the carotid bifurcation, measuring 6.64 × 3.95 cm, with early ECA filling (arrow), turbulent intra-aneurysmal opacification (*), delayed ICA filling (white arrow), and a filling defect compatible with intraluminal thrombosis. **(E–G)** Lateral projections confirming early ECA opacification, turbulent aneurysmal flow, and markedly delayed intracranial perfusion.

Digital subtraction angiography (DSA) demonstrated severe hemodynamic compromise associated with the giant left cervical ICA aneurysm. Contrast preferentially opacified the external carotid artery (ECA), with marked delay in filling of the left ICA and reduced antegrade flow to the intracranial segments. In addition, the aneurysm contained a partially thrombosed component with turbulent intra-aneurysmal flow, raising the possibility of concomitant thromboembolic phenomena. Collateral circulation was identified via the contralateral ICA and ipsilateral ECA branches. The lesion was identified as a giant, partially thrombosed cervical ICA aneurysm arising near the common carotid artery bifurcation. Overall imaging features were considered suggestive of an underlying dissecting arteriopathy (Figure 3). Given the size, mass effect, and hemodynamic compromise caused by the left cervical ICA aneurysm, surgical intervention was proposed to exclude the lesion from the circulation, relieve cervical mass effect, and stabilize cerebral perfusion. Cardiology consultation found no evidence of hypoperfusion, hemodynamic instability, or impending rupture associated with the aortic dissection, and no contraindication to neurosurgical intervention was identified.

Two surgical strategies were considered: aneurysm exclusion with aneurysmectomy and trapping with extracranial–intracranial bypass. An initial cervical exploration allowed exposure of the aneurysm dome; however, the aneurysm neck/base could not be clearly identified due to limited operative space, difficult mobilization, and dense adherence to the distal common carotid artery (CCA), proximal ICA, and ECA. No vascular reconstruction could be safely performed at this stage. A second surgery was planned with the intention of performing a high-flow extracranial–intracranial bypass. At that stage, the precise anatomical relationship between the aneurysm and carotid vessels remained uncertain. Therefore, high-flow bypass was considered necessary to ensure cerebral perfusion if parent vessel sacrifice or complex carotid reconstruction became unavoidable.

The first-stage exploration nevertheless provided critical anatomical information. Although definitive treatment could not be safely performed because the aneurysm neck and its relationship to the carotid bifurcation could not be adequately identified, the dissection clarified the overall orientation of the lesion and facilitated subsequent operative planning.

Based on the improved anatomical understanding obtained during prior dissection, a definitive cervical approach was performed. Re-exploration revealed a 2-cm aneurysm neck arising from the lateral aspect of the left CCA near its bifurcation, with extension toward the origin of the internal carotid artery. After proximal and distal vascular control was achieved, the aneurysm neck was occluded with a vascular clamp. A puncture test confirmed absence of inflow, after which the aneurysm sac was opened and debulked to facilitate dissection from surrounding structures. Complete aneurysmectomy was performed, maintaining temporary vascular control, the aneurysm neck was secured with double 1-0 silk ligation and divided. The common carotid artery and external carotid artery were preserved. The proximal internal carotid artery was found to be chronically thrombosed and therefore no carotid reconstruction was performed. Doppler ultrasonography and fluorescein angiography confirmed preservation of flow through the common and external carotid arteries. Identification of the aneurysm neck while preserving the carotid bifurcation fundamentally altered the operative strategy. This anatomical configuration made aneurysm exclusion without carotid reconstruction technically feasible. Combined with qualitative angiographic evidence of collateral circulation and intraoperative vascular assessment, these findings supported proceeding without immediate cerebral revascularization.

**Figure 4:**
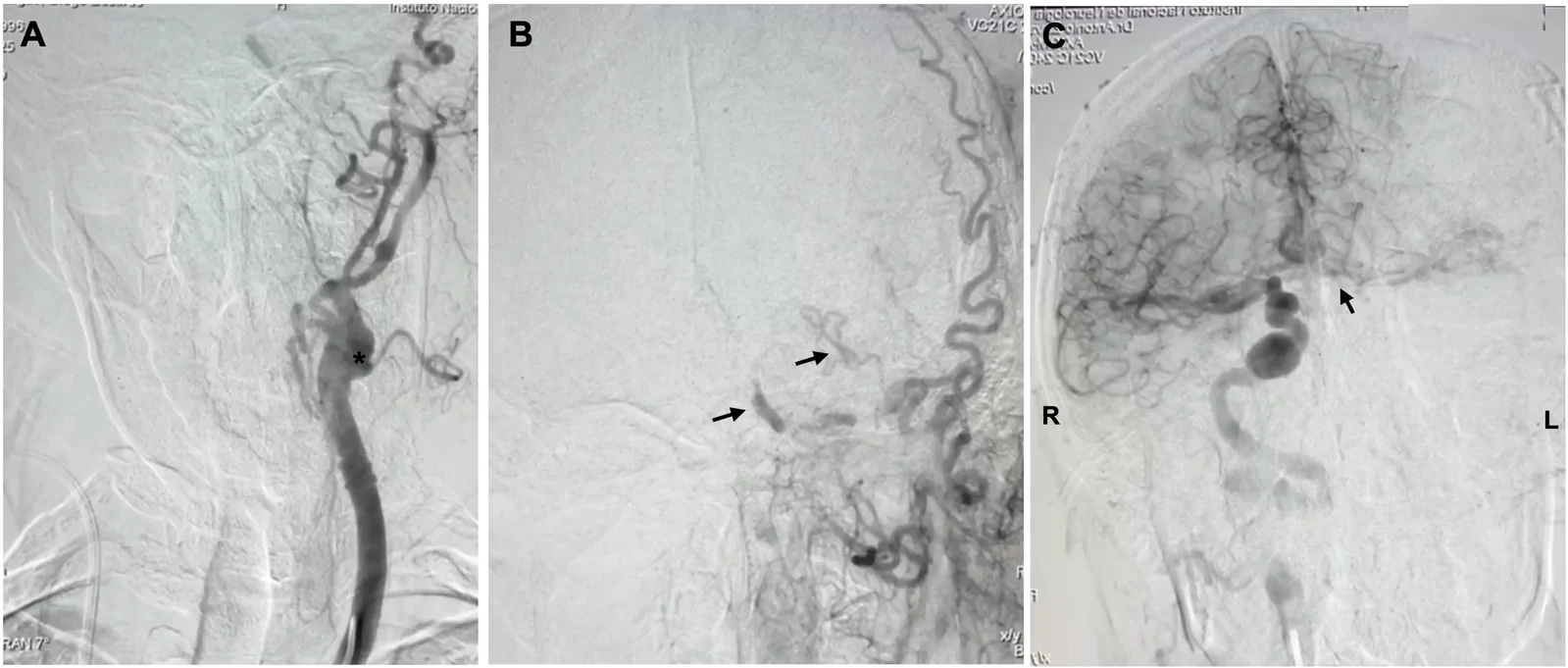
Postoperative DSA. **(A)** A focal residual enlargement of the carotid artery is observed at the site of aneurysm resection (*), without contrast extravasation. **(B,C)** The left intracranial ICA showed limited antegrade filling, while collateral hemispheric perfusion is observed principally by circulation through ethmoidal branches of the ECA, the contralateral ICA—mainly via the anterior communicating artery—and additional ECA-to-intracranial collaterals (arrows). No major contribution from the posterior circulation was identified.

The postoperative course was uneventful. The patient demonstrated improvement in language output, fluency, and comprehension. A follow-up DSA performed on postoperative day 4 confirmed complete exclusion of the aneurysm (Figure 4). No new ischemic events were observed. The patient was discharged on postoperative day 5, neurologically stable, with improved but persistent aphasia and right hemiparesis. At the two-week follow-up, motor strength had improved to 3/5 compared with 1/5 at initial presentation. Antiplatelet therapy and analgesics were prescribed, and outpatient neurosurgical follow-up was arranged. Given the patient's young age and extensive multifocal aneurysmal disease, referral for genetic evaluation and investigation of a possible hereditary systemic arteriopathy was initiated. However, comprehensive molecular testing had not yet been performed at the time of manuscript preparation because these studies were not routinely available in our public healthcare system and required external evaluation through private laboratories.

## Discussion

Extracranial carotid artery aneurysms are an uncommon vascular condition, accounting for less than 1% of all arterial aneurysms and up to 2%–3% of peripheral aneurysms in reported series (1–3). Owing to their rarity, available evidence is largely derived from case reports and small series, precluding standardized management guidelines and necessitating individualized therapeutic strategies. In young adults, these aneurysms are predominantly non-atherosclerotic in origin, with arterial dissection representing the most frequent underlying mechanism (6, 7). Additional etiologies include connective tissue disorders, fibromuscular dysplasia, infectious causes, trauma-related pseudoaneurysms, and iatrogenic injury, frequently associated with multifocal aneurysmal disease and arterial tortuosity (3, 8). Despite comprehensive evaluation, a subset of patients remains without an identifiable cause, underscoring the importance of etiological recognition for management decisions, long-term surveillance, and family counseling. These lesions may also produce complex cerebral hemodynamic alterations and thromboembolic phenomena, particularly when giant size, partial thrombosis, and chronic flow limitation coexist.

The coexistence of aneurysms across intracranial, extracranial, and aortic territories has been associated with systemic vascular disease rather than isolated focal pathology (3, 9, 10). Epidemiological studies have consistently demonstrated a higher prevalence of intracranial aneurysms in patients with aortic aneurysms and, conversely, an increased frequency of extracranial aneurysms in patients with multiple intracranial aneurysms (9–11). These associations are supported by shared structural and molecular mechanisms, including extracellular matrix degeneration, medial smooth muscle cell loss, chronic inflammation, and dysregulation of signaling pathways implicated in heritable arteriopathies. Recognition of this systemic pattern has direct clinical implications, enabling improved risk stratification, more comprehensive vascular screening, and safer therapeutic decision-making beyond management focused solely on the initially identified lesion.

Regardless of etiology, cerebral embolization remains the principal risk associated, promoted by mural thrombus, ulceration, and turbulent flow, while rupture is less common and typically confined to infected or rapidly expanding lesions (1, 3, 8). From a diagnostic standpoint, Doppler ultrasonography remains a useful first-line tool due to its accessibility and ability to detect intraluminal thrombus; however, CT angiography and DSA are essential for comprehensive assessment of vascular anatomy, collateral circulation, and therapeutic planning (12).

In the present case, the exact mechanism underlying the ischemic presentation could not be definitively established. Although angiography demonstrated marked flow delay and severe hemodynamic compromise related to the giant cervical aneurysm, the presence of partial thrombosis and turbulent intra-aneurysmal flow also suggests a possible artery-to-artery thromboembolic component. Therefore, the ischemic stroke was likely multifactorial, involving both chronic hemodynamic insufficiency and embolic mechanisms associated with the thrombosed aneurysm.

Management must be individualized. Open surgery remains the preferred option when anatomy allows, enabling aneurysm resection and arterial reconstruction (1, 4, 5). In complex ICA aneurysms, the need for cerebral revascularization should be individualized according to anatomical characteristics, collateral circulation, hemodynamic considerations, and the anticipated consequences of parent vessel exclusion. Although bypass may be beneficial in selected patients, the optimal indications remain case-dependent and should be interpreted within the context of the overall vascular and clinical scenario (13).

In our patient, the chronic progressive nature of the lesion may also have facilitated adaptive collateral recruitment over time. Angiographic evaluation demonstrated effective collateral flow and preserved perfusion following aneurysm exclusion, supporting a non-revascularization strategy. The decision to avoid extracranial–intracranial bypass was based on qualitative angiographic evidence of hemispheric collateral perfusion, preserved carotid circulation following aneurysm trapping, intraoperative Doppler ultrasonography, fluorescein angiography confirming vessel patency, and the absence of additional ischemic deterioration during the postoperative course. Postoperative angiography demonstrated complete aneurysm exclusion, preservation of carotid circulation, and sustained hemispheric perfusion through established collateral pathways, supporting the hemodynamic adequacy of the chosen strategy during short-term follow-up.

Careful preoperative angiographic assessment and hemodynamic evaluation are therefore essential to guide revascularization decisions and minimize unnecessary bypass-related morbidity, particularly in patients with bilateral carotid artery disease. However, the present case should not be interpreted as establishing definitive criteria for bypass indication, given the qualitative nature of the hemodynamic assessment and the limited follow-up available.

Although endovascular options have expanded the therapeutic armamentarium for extracranial carotid aneurysms, feasibility is highly dependent on vascular anatomy and lesion morphology. Contemporary series and reviews report higher technical failure rates and early stent-related complications in markedly tortuous carotid arteries, as well as challenges related to device navigation and adequate stent deployment in hostile anatomy (14). In addition, the presence of intraluminal thrombus raises embolic concerns and may be associated with incomplete durable exclusion or persistent mass effect when purely endovascular strategies are employed, supporting consideration of open surgery (15). In the present case, the combination of giant size, partial thrombosis and diffuse carotid dolichoectasia favored an open surgical approach to achieve definitive aneurysm exclusion and immediate direct decompression, factors that were particularly relevant in this complex hemodynamic scenario.

Despite the favorable short-term postoperative evolution observed in the present case, the patient remains at risk for progression of the underlying systemic arteriopathy, enlargement of residual aneurysms, and development of additional vascular lesions. Long-term neurovascular and systemic vascular surveillance therefore remains essential.

## Limitations

This report has several limitations. First, the follow-up period remains relatively short, limiting assessment of long-term aneurysm progression, recurrence, and durability of the hemodynamic adaptation after aneurysm exclusion. Second, although the imaging and clinical features suggested an underlying dissecting or systemic arteriopathic process, definitive etiological characterization was not available. Comprehensive genetic testing had not yet been performed at the time of manuscript preparation because these studies were not routinely available in our public healthcare system and required external evaluation through private laboratories. Therefore, the underlying systemic arteriopathy remains presumptive, and conclusions regarding disease pathogenesis should be interpreted with caution. Third, the decision to avoid extracranial–intracranial bypass was based primarily on qualitative angiographic and intraoperative assessment of collateral circulation and cerebral perfusion. Formal balloon test occlusion, quantitative perfusion imaging, and neurophysiological monitoring were not performed. These limitations should be considered when interpreting the surgical strategy and clinical outcome described in this case.

## Patient perspective

When I was admitted to the hospital, I suddenly lost my ability to speak and move the right side of my body. I was frightened because I did not know the cause of my symptoms. The medical team explained that I had a rare vascular condition involving a large aneurysm in my neck and that the surgery would be complex. Although more than one operation was required, I understood that this approach was necessary to perform the procedure as safely as possible.

After surgery, I gradually noticed improvement in my speech and strength, although I am still recovering. I am grateful for the care I received and understand that I will require long-term follow-up because of the remaining vascular abnormalities. I hope that sharing my experience may help physicians better understand and treat patients with similar rare conditions.

## Conclusions

The coexistence of intracranial and extracranial aneurysms may reflect an underlying systemic vascular disorder requiring comprehensive vascular evaluation and long-term surveillance. Decisions regarding cerebral revascularization should be individualized and guided by detailed anatomical and hemodynamic assessment. In selected patients with adequate collateral circulation, aneurysm exclusion without cerebral revascularization may be considered when complex anatomy, partial thrombosis, or vascular tortuosity limit reconstructive or endovascular options. Given the short follow-up and unresolved underlying arteriopathy, continued longitudinal surveillance remains essential.

## Data Availability

The original contributions presented in the study are included in the article/Supplementary Material, further inquiries can be directed to the corresponding author.
